# Short-term improvement of mental health after a COVID-19 vaccination

**DOI:** 10.1371/journal.pone.0280587

**Published:** 2023-02-15

**Authors:** Charilaos Chourpiliadis, Anikó Lovik, Anna K. Kähler, Unnur A. Valdimarsdóttir, Emma M. Frans, Fredrik Nyberg, Patrick F. Sullivan, Fang Fang

**Affiliations:** 1 Institute of Environmental Medicine, Karolinska Institutet, Stockholm, Sweden; 2 Department of Medical Epidemiology and Biostatistics, Karolinska Institutet, Stockholm, Sweden; 3 Centre of Public Health Sciences, Faculty of Medicine, School of Health Sciences, University of Iceland, Reykjavik, Iceland; 4 Department of Epidemiology, Harvard TH Chan School of Public Health, Boston, Massachusetts, United States of America; 5 School of Public Health and Community Medicine, Institute of Medicine, Sahlgrenska Academy, University of Gothenburg, Gothenburg, Sweden; 6 Departments of Genetics and Psychiatry, University of North Carolina, Chapel Hill, North Carolina, United States of America; The University of Texas MD Anderson Cancer Center, UNITED STATES

## Abstract

**Introduction:**

The role of COVID-19 vaccination on the mental health of the general population remains poorly understood. This study aims to assess the short-term change in depressive and anxiety symptoms in relation to COVID-19 vaccination among Swedish adults.

**Methods:**

A prospective study of 7,925 individuals recruited from ongoing cohort studies at the Karolinska Institutet, Stockholm, Sweden, or through social media campaigns, with monthly data collections on self-reported depressive and anxiety symptoms from December 2020 to October 2021 and COVID-19 vaccination from July to October 2021. Prevalence of depressive and anxiety symptoms (defined as a self-reported total score of ≥10 in PHQ-9 and GAD-7, respectively) was calculated one month before, one month after the first dose, and, if applicable, one month after the second dose. For individuals not vaccinated or choosing not to report vaccination status (unvaccinated individuals), we selected three monthly measures of PHQ-9 and GAD-7 with 2-month intervals in-between based on data availability.

**Results:**

5,079 (64.1%) individuals received two doses of COVID-19 vaccine, 1,977 (24.9%) received one dose, 305 (3.9%) were not vaccinated, and 564 (7.1%) chose not to report vaccination status. There was a lower prevalence of depressive and anxiety symptoms among vaccinated, compared to unvaccinated individuals, especially after the second dose. Among individuals receiving two doses of vaccine, the prevalence of depressive and anxiety symptoms was lower after both first (aRR = 0.82, 95%CI 0.76–0.88 for depression; aRR = 0.81, 95%CI 0.73–0.89 for anxiety) and second (aRR = 0.79, 95%CI 0.73–0.85 for depression; aRR = 0.73, 95%CI 0.66–0.81 for anxiety) dose, compared to before vaccination. Similar results were observed among individuals receiving only one dose (aRR = 0.76, 95%CI 0.68–0.84 for depression; aRR = 0.82, 95%CI 0.72–0.94 for anxiety), comparing after first dose to before vaccination.

**Conclusions:**

We observed a short-term improvement in depressive and anxiety symptoms among adults receiving COVID-19 vaccines in the current pandemic. Our findings provide new evidence to support outreach campaigns targeting hesitant groups.

## Introduction

A negative mental health impact of the COVID-19 pandemic has been consistently demonstrated, affecting both the infected and non-infected individuals [1]. For instance, research has shown that individuals affected by the SARS-Cov-2 infection have an increased risk for developing common psychiatric disorders, including mood and anxiety disorders, and that the severity of the infection could modify such risk increase [2, 3]. Neuroinflammation subsequent to COVID-19, especially the severe form of COVID-19, might contribute to the increased risk of psychiatric disorders among the affected indivdiuals, as neuroinflammation can for instance lead to mental fatigue and mood alterations [4]. An increased burden of psychiatric symptoms has also been reported among individuals not directly affected by COVID-19, likely attributable to multiple factors related to mitigating measures (e.g., lockdown and quarantine), and the fear of being infected or infecting others [5, 6]. Vaccination against COVID-19 has, on the other hand, been suggested to have a beneficial impact on mental health during the pandemic, in addition to protection from severe COVID-19 [7]. Previous studies have indeed provided some evidence that vaccinated individuals felt more protected against COVID-19 and reported lower burden of psychiatric symptoms [7–10]. However, the existing studies rest on a restricted phase of the vaccine rollout [7, 9, 10] with relatively small sample size [8, 11].

Our aim was to provide an estimate of the short-term change in mental health after the vaccination against COVID-19, specifically in terms of self-reported symptoms of depression and anxiety, during a long phase of the vaccine rollout and using a large study sample with careful adjustment for the impact of age, sex, body mass index (BMI), relationship status, smoking, number of physical comorbidities, history of psychiatric disorders, history of COVID-19 infection, as well as calendar time and type of recruitment to the study. We hypothesized that receiving a COVID-19 vaccine was associated with a decrease in the prevalence of self-reported symptoms of depression and anxiety.

## Methods

### Participants

We performed a longitudinal study based on the Omtanke2020 study, which is a closed cohort study with an enrolment period from June 2020 to June 2021 and specifically designed to assess the mental health impact of COVID-19 in Sweden [12]. Eligible to participate in the Omtanke2020 study were all adults residing in Sweden who could have access to electronic identification using BankID and could understand Swedish. The participants were recruited either with personal invitations from ongoing studies at Karolinska Institutet (LifeGene, KARMA, and Swedish Twin Registry) or self-recruited from social media campaigns. A total of 28,293 adults residing in Sweden were recruited to Omtanke2020 during the enrolment period and provided (will provide) self-reported data on mental and physical health, sociodemographic factors, lifestyle factors, and COVID-19-specific questions such as infection and vaccination at baseline, 11 monthly follow-ups, and five annual follow-ups. There is a relatively large rate of loss to follow-up. For instance, among all participants recruited to the study, only 18,101 (64%) completed the first annual follow-up during December-January 2022. This is partly because the Omtanke2020 participants are able to withdraw any time they wish and ask for the removal of their data.

### Procedure

In the present study, all participants of Omtanke2020 with monthly self-reported data on depressive and anxiety symptoms between December 2020 and October 2021 were deemed eligible. These participants also had to have data on COVID-19 vaccination collected between 17^th^ July and 30^th^ October 2021. However, not everyone had available data on depression and anxiety for every survey month. Thus, for vaccinated individuals, we included only those individuals with measurements of depressive or anxiety symptoms one month before the first dose, one month after the first dose, and, if applicable, one month after the second dose. Among individuals with two doses, the average (±standard deviation) time interval between the two doses was 1.6±0.6 months. For unvaccinated individuals, we included those with at least one set of three consecutive depression or anxiety measurements two months apart during the study period (i.e., baseline/Time 0, 2 months after baseline/Time 1, and 4 months after baseline/Time 2). For those with more than three consecutive measurements one set of such three measurements was selected randomly. We excluded from the analysis 40 participants with inconsistent information on vaccination (e.g., time of the 2^nd^ dose preceding that of the 1^st^ dose) and 22 participants who received the 1-dose Ad26.COV2.S (Janssen vaccine). We defined the vaccinated individuals as those reporting having received two doses or one dose of a COVID-19 vaccine and the unvaccinated individuals as those reporting no vaccination or chose not to report vaccination status. A schematic diagram of inclusion and exclusion criteria can be found in S1 File. In the main analysis, we classified individuals choosing not to report vaccination status as unvaccinated, assuming that individuals that received vaccination would be more willing to report vaccination status than individuals that did not receive vaccination. To assess the validity of this assumption, we performed sensitivity analysis to analyze separately individuals reporting no vaccination and individuals choosing not to report vaccination status.

### Measures (questionnaires)

The Patient Health Questionnaire (PHQ-9) [13] was used to measure depressive symptoms, whereas the Generalized Anxiety Disorder (GAD-7) [14] scale was used to measure anxiety symptoms. We first calculated the total scores of both scales, which are shown to have appropriate psychometric properties in Omtanke2020 [12]. As a cut-off of 10 has previously been established to distinguish with optimal sensitivity and specificity mild from severe cases of major depression as well as generalized anxiety disorder [13, 14], we used a cut-off of 10 on the total score of each scale to define above-threshold, self-reported, symptoms of depression and anxiety, respectively.

### Statistical analysis

Poisson regression models with robust sandwich estimator were used to calculate the prevalence ratio, comparing the prevalence of self-reported depressive or anxiety symptoms between vaccinated and unvaccinated individuals cross-sectionally, at baseline, after 1^st^ dose/Time 1, and after 2^nd^ dose/Time 2. The robust sandwich estimator approach has been implemented to account for the overestimation of standard error when using Poisson regression models with binary data [15, 16]. To disentangle the seasonality effect from the vaccine effect, we performed a stratified analysis by calendar month. We first calculated the crude prevalence ratio and then adjusted the analysis for age (18–29, 30–39, 40–49, 50–59, 60–69, or 70+), sex (men or women), body mass index (BMI; <18.5 underweight, 18.5–25 normal weight, >25–30 overweight, or >30 obese), recruitment type (self-recruitment or by invitation), relationship status (single or in a relationship), current smoking (yes or no), number of physical comorbidities (1, 2, or ≥3), history of psychiatric disorder (yes or no), history of COVID-19 infection (not infected, infected but not hospitalized, or infected and hospitalized), and the month of the baseline survey. The covariates selection was determined by previous literature and the availability of information from the Omtanke2020 study [8, 10, 12]. Some of the covariates (e.g., recruitment type) are relevant to the assessment of potential selection bias [17], whereas others are related to mental health [3, 18–20] or both mental health and vaccination status [19, 21].

We also used a Generalized Estimating Equations (GEE) model to assess the temporal change in the prevalence of self-reported depressive and anxiety symptoms among vaccinated and unvaccinated individuals separately. The predictor was linked to the data through the logit of the risk with Poisson distribution, and unstructured correlation was assumed. Estimates of standard errors were produced with the robust sandwich estimator.

The statistical significance level was reported at alpha = 0.05. Missingness of covariates ranged from 0.1–5%, and complete case analysis was considered for all the analyses. Statistical analyses were performed using STATA 16 (College Station, TX: StataCorp LLC).

The study was approved by the Swedish Ethical Review Authority on 3 June 2020 (DNR 2020–01785).

## Results

A total of 7,925 individuals, with a mean (±SD) age of 52.6 (±15.4) years and 83% women, were included in the analysis, including 5,079 (64.09%) with two doses and 1,977 (24.95%) with one dose of COVID-19 vaccine, 305 (3.85%) not vaccinated, and 564 (7.12%) whose vaccination status was not reported (Table 1).

**Table 1:** Baseline characteristics of the study participants by vaccination status.

	All participants	Two doses of vaccine	One dose of vaccine	Not vaccinated	Vaccination status not reported
No. (%)	7925 (100%)	5079 (64.09%)	1977 (24.95%)	305 (3.85%)	564 (7.11%)
**Age (years), No. (%)**						
18–29	698 (8.81%)	412 (8.11%)	199 (10.12%)	36 (11.80%)	51 (9.04%)
30–39	1110 (14.01%)	681 (13.41%)	293 (14.82%)	56 (18.36%)	80 (14.18%)
40–49	1411 (17.80%)	811 (15.97%)	390 (19.73%)	71 (23.28%)	139 (24.65%)
50–59	1806 (22.79%)	1080 (21.26%)	524 (26.50%)	71 (23.28%)	131 (23.23%)
60–69	1601 (20.20%)	1100 (21.66%)	360 (18.21%)	50 (16.39%)	91 (16.13%)
70+	1299 (16.39%)	995 (19.59%)	211 (10.67%)	21 (6.89%)	72 (12.77%)
**Sex, No. (%)**						
Men	1350 (17.03%)	925 (18.21%)	285 (14.42%)	41 (13.44%)	99 (17.55%)
Women	6575 (82.97%)	4154 (81.79%)	1692 (85.58%)	264 (86.56%)	465 (82.45%)
**Recruitment type, No. (%)**						
By invitation	4325 (54.57%)	3068 (60.41%)	850 (42.99%)	149 (48.85%)	258 (45.74%)
Self-recruitment	3600 (45.43%)	2011 (39.59%)	1127 (57.01%)	156 (51.15%)	306 (54.26%)
**Body mass index (Kg/m** ^**2**^ **), No. (%)**						
<18.5, underweight	121 (1.53%)	71 (1.40%)	36 (1.82%)	6 (1.97%)	8 (1.42%)
18.5–25, normal weight	4190 (52.87%)	2727 (53.69%)	1016 (51.39%)	156 (51.15%)	291 (51.60%)
>25–30, overweight	2333 (29.44%)	1504 (29.61%)	575 (29.08%)	93 (30.49%)	161 (28.55%)
>30, obese	943 (11.90%)	567 (11.16%)	257 (13%)	35 (11.48%)	84 (14.89%)
Missing	338 (4.26%)	210 (4.13%)	93 (4.70%)	15 (4.92%)	20 (3.55%)
**Number of comorbidities, No. (%)**						
None	5338(67.36%)	3391 (66.77%)	1363 (68.94%)	208 (68.20%)	376 (66.67%)
1	1910 (24.1%)	1225 (24.12%)	469 (23.72%)	77 (25.25%)	139 (24.65%)
2	491 (6.20%)	332 (6.54%)	110 (5.56%)	13 (4.26%)	36 (6.38%)
≥3	167 (2.11%)	118 (2.32%)	33 (1.67%)	7 (2.30%)	9 (1.60%)
Missing	19 (0.24%)	13 (0.26%)	2 (0.10%)	0 (0.00%)	4 (0.71%)
**History of psychiatric disorder, No. (%)**						
No	5480(69.15%)	3644 (71.75%)	1288 (65.15%)	184 (60.33%)	364 (64.54%)
Yes	2352 (29.6%)	1375 (27.07%)	673 (34.04%)	117 (38.36%)	187 (33.16%)
Missing	93 (1.17%)	60 (1.18%)	16 (0.81%)	4 (1.31%)	13 (2.30%)
**Current smoking, No. (%)**						
No	6901 (87.08%)	4468 (87.97%)	1698 (85.89%)	259 (84.92%)	476 (84.40%)
Yes	989 (12.48%)	588 (11.58%)	274 (13.86%)	44 (14.43%)	83 (14.72%)
Missing	35 (0.44%)	23 (0.45%)	5 (0.25%)	2 (0.66%)	5 (0.89%)
**Relationship status, No. (%)**						
Single	2178 (27.48%)	1361 (26.80%)	579 (29.29%)	97 (31.80%)	141 (25.00%)
In a relationship	5714 (72.10%)	3703 (72.91%)	1385 (70.06%)	206 (67.54%)	420 (74.47%)
Missing	33 (0.42%)	15 (0.30%)	13 (0.66%)	2 (0.66%)	3 (0.53%)
**History of COVID-19 infection, No. (%)**						
Not infected	4183 (52.78%)	2732 (53.79%)	1047 (52.96%)	155 (50.82%)	249 (44.15%)
Infected,not hospitalized	3340 (42.15%)	2106 (41.46%)	817 (41.33%)	134 (43.93%)	283 (50.18%)
Infected, hospitalized	402 (5.07%)	241 (4.75%)	113 (5.72%)	16 (5.25%)	32 (5.67%)

Although there tended to be a lower prevalence of self-reported depressive and anxiety symptoms among the vaccinated individuals, compared with the unvaccinated, already at baseline, the difference tended to become greater after the 2^nd^ dose among those with two doses of vaccine and after the 1^st^ dose among those with one dose of vaccine (Table 2). None of these differences were however statistically significant given the overlapping confidence intervals between prevalence ratios. The stratified analysis by calendar month showed a similar result pattern across different calendar months (S1 Table). A similar pattern of results was also observed when exploring the association among those reporting no vaccination or choosing not to report vaccination status (S2 Table).

**Table 2:** Prevalence ratio (PR) and 95% confidence interval (CI) of self-reported depressive and anxiety symptoms comparing vaccinated to unvaccinated individuals.

***Depression***				
	**Prevalence in unvaccinated individuals; N (%)**	**Prevalence in individuals with 2 doses; N (%)**	**Crude PR (95%CI)**	Adjusted PR (95%CI)*
**Before 1**^**st**^ **dose or baseline**	182 (20.9%)	675 (13.3%)	0.64 (0.55–0.74)	0.74 (0.61–0.90)
**After 1**^**st**^ **dose or 2 months after baseline**	173 (19.9%)	551 (10.8%)	0.55 (0.47–0.64)	0.77 (0.62–0.96)
**After 2**^**nd**^ **dose or 4 months after baseline**	157 (18.1%)	534 (10.5%)	0.58 (0.45–0.69)	0.65 (0.52–0.81)
	**Prevalence in unvaccinated individuals; N (%)**	**Prevalence in individuals with 1 dose; N (%)**	**Crude PR (95%CI)**	**Adjusted PR (95%CI)+**
**Before 1**^**st**^ **dose or baseline**	182 (20.9%)	343 (17.4%)	0.83 (0.71–0.97)	0.86 (0.67–1.10)
**After 1**^**st**^ **dose or 2 months after baseline**	173 (19.9%)	262 (13.3%)	0.67 (0.56–0.79)	0.71 (0.53–0.95)
***Anxiety***				
	**Prevalence in unvaccinated individuals; N (%)**	**Prevalence in individuals with 2 doses; N (%)**	**Crude PR (95%CI)**	**Adjusted PR (95%CI)** *
**Before 1**^**st**^ **dose or baseline**	103 (11.9%)	466 (9.2%)	0.77 (0.63–0.95)	0.76 (0.58–1.00)
**After 1**^**st**^ **dose or 2 months after baseline**	123 (14.2%)	382 (7.5%)	0.53 (0.44–0.64)	0.69 (0.52–0.91)
**After 2**^**nd**^ **dose or 4 months after baseline**	106 (12.2%)	343 (6.7%)	0.55 (0.45–0.68)	0.59 (0.44–0.81)
	**Prevalence in unvaccinated individuals; N (%)**	**Prevalence in individuals with 1 dose; N (%)**	**Crude PR (95%CI)**	**Adjusted PR (95%CI)+**
**Before 1**^**st**^ **dose or baseline**	103 (11.9%)	233 (11.8%)	0.99 (0.80–1.24)	1.10 (0.76–1.58)
**After 1**^**st**^ **dose or 2 months after baseline**	123 (14.2%)	186 (9.4%)	0.67 (0.537–0.823)	0.75 (0.52–1.06)

Analysis was performed using Poisson regression and adjusted for age, sex, recruitment type, body mass index, relationship status, current smoking, number of comorbidities, history of psychiatric disorder, history of COVID-19 infection, and month of baseline survey.

*The (complete-case) analysis of adjusted PR included 817 unvaccinated and 4,807 vaccinated individuals with two vaccine doses.

+The (complete-case) analysis of adjusted PR included 817 unvaccinated and 1,858 vaccinated individuals with one vaccine dose.

**Fig 1:**
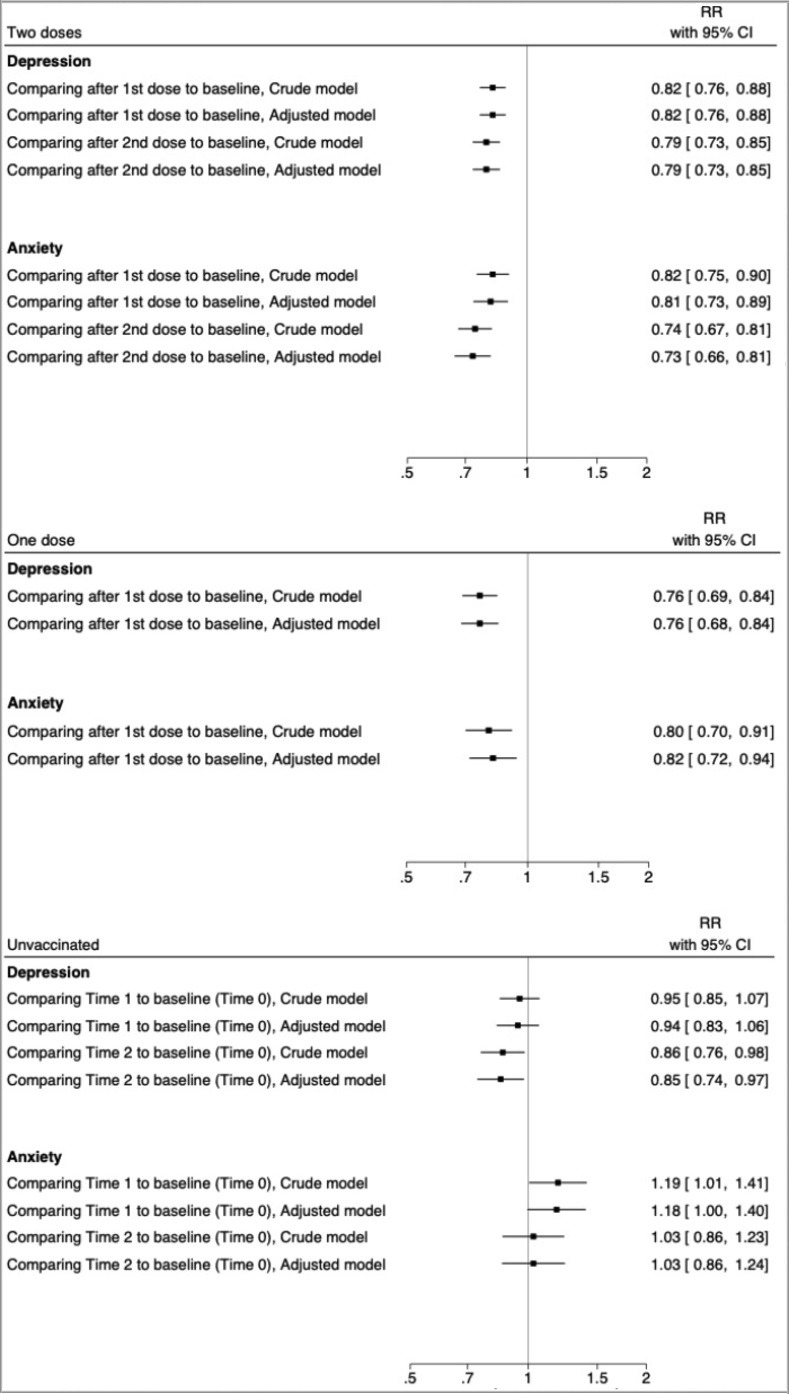
Risk Ratio comparing the prevalence of self-reported depressive and anxiety symptoms after to before vaccination. Risk Ratio (RR) with 95% confidence interval (CI) of depression and anxiety, comparing the prevalence of self-reported depressive and anxiety symptoms after COVID-19 vaccine (1^st^ or 2^nd^ dose) to the prevalence before vaccine among vaccinated individuals, and comparing the prevalence of later time points to baseline among the unvaccinated individuals. Unvaccinated individuals include individuals reporting no vaccination or choosing not to report vaccination status. The analyses were performed using GEE models and adjusted for age, sex, recruitment type, body mass index, relationship status, current smoking, number of comorbidities, history of psychiatric disorder, history of COVID-19 infection, and month of baseline survey. The risk ratio is plotted as a box, and the 95% confidence interval as the line.

Fig 1 shows the risk ratio (RR) of depression and anxiety, comparing the prevalence of self-reported depressive and anxiety symptoms after a COVID-19 vaccine (1^st^ or 2^nd^ dose) to the prevalence before vaccination among the vaccinated individuals or the prevalence at later time points (Time 1 or 2) to the prevalence at the first time point (Time 0) among the unvaccinated individuals. There was a decline in the prevalence of self-reported depressive and anxiety symptoms among individuals that received two doses of vaccine, both one month after 1^st^ dose (aRR = 0.82; 95%CI 0.76–0.88 for depression; aRR = 0.81; 95%CI 0.73–0.89 for anxiety) and one month after 2^nd^ dose (aRR = 0.79; 95%CI 0.73–0.85 for depression; aRR = 0.73; 95%CI 0.66–0.81 for anxiety). A decrease in prevalence was also noted one month after the 1^st^ dose among individuals receiving only one vaccine dose (aRR = 0.76; 95CI 0.68–0.84 for depression; aRR = 0.82; 95%CI 0.72–0.94 for anxiety). Among the unvaccinated individuals, there was no statistically significant decrease in the prevalence of self-reported depressive or anxiety symptoms over time, except when comparing the prevalence of depressive symptoms at Time 2 to that of Time 0. Similar results were produced among those reporting no vaccination but not among those choosing not to report vaccination status (S1 Fig).

## Discussion

The present study demonstrated a short-term improvement in the prevalence of self-reported depressive and anxiety symptoms after COVID-19 vaccination in a large sample of Swedish adults. The improvement was more pronounced after the second dose, compared with the first dose, independent of age, sex, body mass index (BMI), recruitment type, relationship status, current smoking, number of comorbidities, history of psychiatric disorder, history of COVID-19 infection, and calendar month.

Our results are consistent with previous research suggesting a positive influence of COVID-19 vaccination on mental health [7–11]. The present study extends, however, the existing knowledge by addressing several methodological issues of previous studies, such as cross-sectional study design [8, 10, 11], relatively small sample size [8], the use of a single mental health instrument (e.g., PHQ-4) [7, 9, 11], and addressing a restricted phase of vaccination, e.g., March 2021 [7] or June 2021 [9, 10]. First, our study used a longitudinal design with monthly surveys on vaccination status and mental health, which allowed us to understand the effect of COVID-19 vaccines on the temporal change of depression and anxiety as well as to disentangle the effect of vaccine from seasonal variation in the prevalence of depression and anxiety. The large sample size, the study of both depression and anxiety using well-established instruments, and the relatively long duration of the study period are other strengths. Collectively, the existing knowledge, including findings of the present study, suggests the importance of COVID-19 vaccination, to not only prevent severe COVID-19 illness but also to improve mental health.

Limitations of the study include self-reported data on COVID-19 vaccination and depressive and anxiety symptoms, leading to a potential concern on misclassification of the exposure and outcomes. The recruitment of study participants through either personal invitation from existing studies or social media campaigns is another limitation as participants recruited through such mechanisms might have different statuses concerning both COVID-19 vaccination and mental health outcomes, compared to the source population, leading therefore to potential selection bias. There might be sources of unmeasured confounding that were not accounted for in the analysis because such information was not available. For example, socioeconomic disparities have been associated with vaccine hesitancy and mental disorders [22] and were not adjusted for in the present study. Further, we observed a higher prevalence of self-reported depressive and anxiety symptoms among unvaccinated participants, compared to vaccinated individuals, at baseline, in accordance with a previous study [23]. Such difference persisted after multivariable adjustment including history of psychiatric disorder and number of somatic diseases, indicating that some degree of residual confounding exists still. Although the findings of the present study add support to the hypothesis that receiving a COVID-19 vaccine is protective against depression and anxiety during the current pandemic, there might be other contributors. For example, as the burden of pandemic (i.e., number of newly diagnosed COVID-19 cases and deaths) and the corresponding mitigating strategies varied substantially during the study period, our emotional reaction toward the pandemic was also expected to vary accordingly. We tried to alleviate this concern by studying depressive and anxiety symptoms within one month after receiving a COVID-19 vaccine, hypothesizing that the pandemic and its mitigating measures were relatively stable in these short time windows. In addition, we adjusted for the month of baseline in all analyses and performed a stratified analysis by the month of baseline to further alleviate this concern. Finally, whether our results are generalizable to other populations remains to be studied due to the largely different burden of the pandemic and population coverage of COVID-19 vaccination across the globe.

## Conclusion

We found an immediate positive change in symptoms of depression and anxiety after receiving a COVID-19 vaccination among adults in the current pandemic. This study has provided new evidence to encourage outreach campaigns targeting vaccine-hesitant groups. A potential benefit would be the increase in the COVID-19 vaccination coverage of the general population.

## Supporting information

S1 File Flowchart based on inclusion and exclusion criteria. (PDF)

S1 Fig Risk ratio comparing the prevalence of depression and anxiety in Time 1 or Time 2 to baseline (Time 0) among those not vaccinated or choosing not to report vaccination status. (TIF)

S1 Table Prevalence (95% confidence interval) of depression and anxiety among vaccinated and unvaccinated individuals by calendar month in 2021. Prevalence was adjusted for age, sex, recruitment type, body mass index, relationship status, current smoking, number of comorbidities, history of psychiatric disorder, and history of COVID-19 infection. Bold font indicates statistically significant difference between vaccinated and unvaccinated individuals. The number of vaccinated individuals includes all those participants who received the first dose of a Covid-19 vaccine during the previous months. Not all participants had monthly information on mental health.
(DOCX)

S2 Table Prevalence ratio (PR) and 95% confidence interval (CI) of depression and anxiety comparing vaccinated (with one or two doses) to those not vaccinated or who chose not to report vaccination status. Analysis was performed using Poisson regression and adjusted for age, sex, recruitment type, body mass index, relationship status, current smoking, number of comorbidities, history of psychiatric disorder, history of COVID-19 infection, and month of baseline survey. *The complete-case analysis of adjusted RR included 284 not vaccinated and 4,807 vaccinated individuals with two doses. +The complete-case analysis of adjusted PR included 284 not vaccinated and 1,858 vaccinated individuals with one dose. **The complete-case analysis of adjusted RR included 533 whose vaccination status was not reported and 4,807 vaccinated individuals with two doses. ++The complete-case analysis of adjusted RR included 533 whose vaccination status was not reported and 1,858 vaccinated individuals with one dose.
(DOCX)
