# Supplementary figures and images for: Short-term improvement of mental health after a COVID-19 vaccination
Source: PLoS One. 2023 Feb 15;18(2):e0280587. doi: 10.1371/journal.pone.0280587 (PMC9931115; doi:10.1371/journal.pone.0280587)

S1 File. Flowchart based on inclusion and exclusion criteria

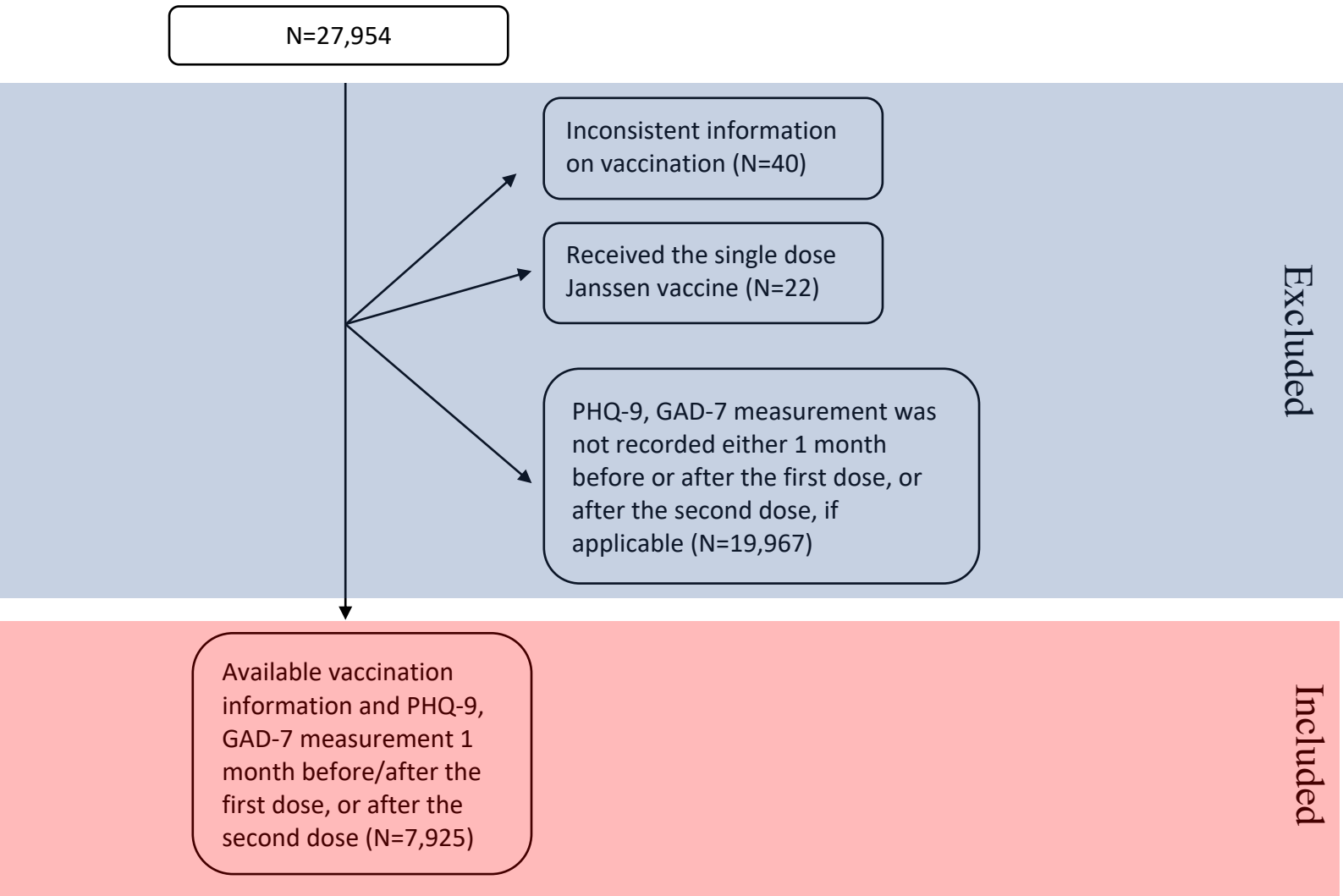

Supplement: S1 File — (PDF) [file pone.0280587.s001.pdf]

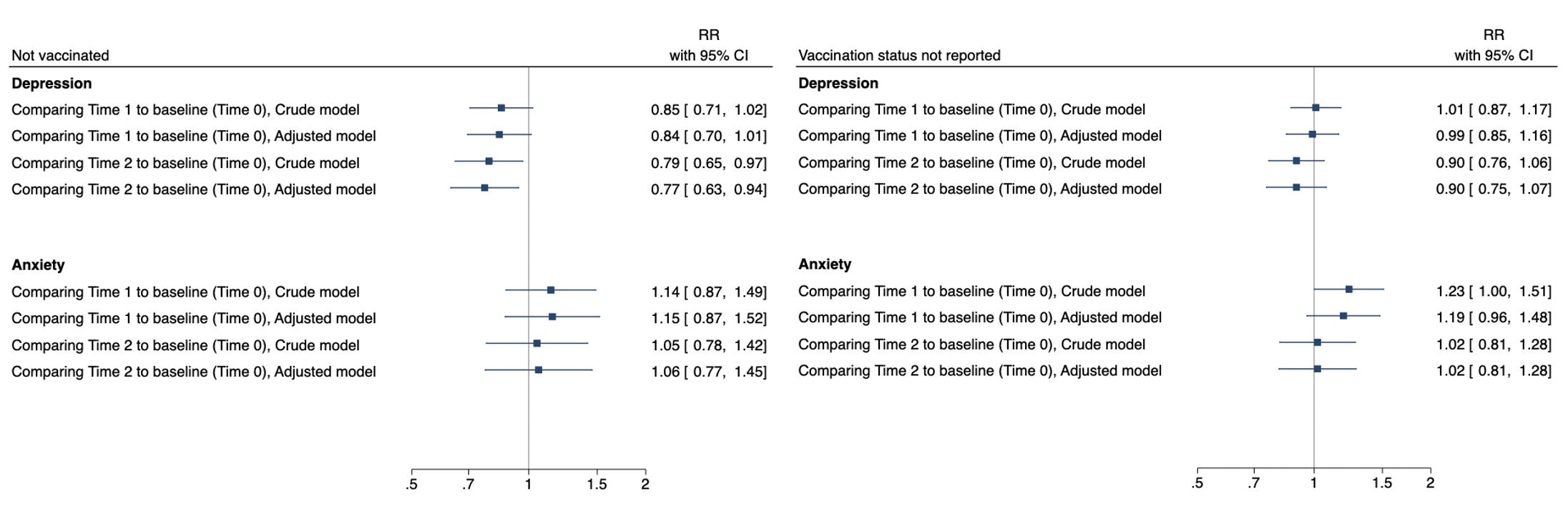

Supplement: S1 Fig — (TIF) [file pone.0280587.s002.tif]
