# Supplementary material for: Short-term improvement of mental health after a COVID-19 vaccination
Source: PLoS One. 2023 Feb 15;18(2):e0280587. doi: 10.1371/journal.pone.0280587 (PMC9931115; doi:10.1371/journal.pone.0280587)
Supplement: S2 Table — Analysis was performed using Poisson regression and adjusted for age, sex, recruitment type, body mass index, relationship status, current smoking, number of comorbidities, history of psychiatric disorder, history of COVID-19 infection, and month of baseline survey. *The complete-case analysis of adjusted RR included 284 not vaccinated and 4,807 vaccinated individuals with two doses. +The complete-case analysis of adjusted PR included 284 not vaccinated and 1,858 vaccinated individuals with one dose. **The complete-case analysis of adjusted RR included 533 whose vaccination status was not reported and 4,807 vaccinated individuals with two doses. ++The complete-case analysis of adjusted RR included 533 whose vaccination status was not reported and 1,858 vaccinated individuals with one dose. (DOCX) [file pone.0280587.s004.docx]

| **S2 Table. Prevalence ratio (PR) and 95% confidence interval (CI) of depression and anxiety comparing vaccinated (with one or doses) to those not vaccinated or who chose not to report vaccination status** | | | | |
| --- | --- | --- | --- | --- |
| ***Depression*** | **Unvaccinated: Not vaccinated** | | | |
|  | **Prevalence in unvaccinated; N (%)** | **Prevalence in individuals with 2 doses; N (%)** | **Crude PR (95%CI)** | **Adjusted PR (95%CI)*** |
| **Baseline** | 68 (22.3%) | 675 (13.3%) | 0.60 (0.48 - 0.74) | 0.73 (0.57 - 0.92) |
| **After 1^st^ dose or 2 months after baseline** | 58 (19%) | 551 (10.8%) | 0.57 (0.45 - 0.73) | 0.81 (0.61 - 1.07) |
| **After 2^nd^ dose or 4 months after baseline** | 54 (17.7%) | 534 (10.5%) | 0.59 (0.46 - 0.77) | 0.70 (0.53 - 0.93) |
|  | **Prevalence in unvaccinated; N (%)** | **Prevalence in individuals with 1 dose; N (%)** | **Crude PR (95%CI)** | **Adjusted PR (95%CI)+** |
| **Baseline** | 68 (22.3%) | 343 (17.4%) | 0.78 (0.62 - 0.98) | 0.87 (0.66 - 1.15) |
| **After 1^st^ dose or 2 months after baseline** | 58 (19%) | 262 (13.3%) | 0.70 (0.54 - 0.90) | 0.72 (0.52 - 1.01) |
|  |  |  |  |  |
| ***Depression*** | **Unvaccinated: Vaccination status not reported** | | | |
|  | **Prevalence in unvaccinated; N (%)** | **Prevalence in individuals with 2 doses; N (%)** | **Crude PR (95%CI)** | **Adjusted PR (95%CI)**** |
| **Baseline** | 114 (20.2%) | 675 (13.3%) | 0.66 (0.55 - 0.79) | 0.76 (0.59 - 0.97) |
| **After 1^st^ dose or 2 months after baseline** | 115 (20.4%) | 551 (10.8%) | 0.53 (0.44 - 0.64) | 0.79 (0.61 - 1.01) |
| **After 2^nd^ dose or 4 months after baseline** | 103 (18.3%) | 534 (10.5%) | 0.58 (0.48 - 0.70) | 0.62 (0.47 - 0.82) |
|  | **Prevalence in unvaccinated; N (%)** | **Prevalence in individuals with 1 dose; N (%)** | **Crude PR (95%CI)** | **Adjusted PR (95%CI)++** |
| **Baseline** | 114 (20.2%) | 343 (17.4%) | 0.86 (0.71 - 1.04) | 0.92 (0.66 - 1.29) |
| **After 1^st^ dose or 2 months after baseline** | 115 (20.4%) | 262 (13.3%) | 0.65 (0.53 - 0.79) | 0.75 (0.51 - 1.10) |
| ***Anxiety*** | **Unvaccinated: Not vaccinated** | | | |
|  | **Prevalence in unvaccinated; N (%)** | **Prevalence in individuals with 2 doses; N (%)** | **Crude PR (95%CI)** | **Adjusted PR (95%CI)*** |
| **Baseline** | 37 (12.1%) | 466 (9.2%) | 0.76 (0.55 - 1.04) | 0.86 (0.61 - 1.22) |
| **After 1^st^ dose or 2 months after baseline** | 42 (13.8%) | 382 (7.5%) | 0.55 (0.41 - 0.74) | 0.75 (0.53 - 1.06) |
| **After 2^nd^ dose or 4 months after baseline** | 39 (12.8%) | 343 (6.7%) | 0.53 (0.39 - 0.72) | 0.62 (0.43 - 0.90) |
|  | **Prevalence in unvaccinated; N (%)** | **Prevalence in individuals with 1 dose; N (%)** | **Crude PR (95%CI)** | **Adjusted PR (95%CI)+** |
| **Baseline** | 37 (12.1%) | 233 (11.8%) | 0.97 (0.70 - 1.35 | 1.33 (0.86 - 2.04) |
| **After 1^st^ dose or 2 months after baseline** | 42 (13.8%) | 186 (9.4%) | 0.68 (0.50 - 0.93 | 0.78 (0.52 - 1.17) |
| ***Anxiety*** | **Unvaccinated: Vaccination status not reported** | | | |
|  | **Prevalence in unvaccinated; N (%)** | **Prevalence in individuals with 2 doses; N (%)** | **Crude PR (95%CI)** | **Adjusted PR (95%CI)**** |
| **Baseline** | 66 (11.7%) | 466 (9.2%) | 0.78 (0.62 - 1.00 | 0.67 (0.46 - 0.97) |
| **After 1^st^ dose or 2 months after baseline** | 82 (14.4%) | 382 (7.5%) | 0.52 (0.42 - 0.65) | 0.64 (0.46 - 0.91) |
| **After 2^nd^ dose or 4 months after baseline** | 67 (11.9%) | 343 (6.7%) | 0.57 (0.44 - 0.73) | 0.59 (0.40 - 0.88) |
|  | **Prevalence in unvaccinated; N (%)** | **Prevalence in individuals with 1 dose; N (%)** | **Crude PR (95%CI)** | **Adjusted PR (95%CI)++** |
| **Baseline** | 66 (11.7%) | 233 (11.8%) | 1.01 (0.78 - 1.30) | 1.03 (0.64 - 1.66) |
| **After 1^st^ dose or 2 months after baseline** | 81 (14.4%) | 186 (9.4%) | 0.66 (0.51 - 0.84) | 0.65 (0.40 - 1.07) |
| Analysis was performed using Poisson regression and adjusting for age, sex, recruitment type, body mass index, relationship status, current smoking, number of comorbidities, history of psychiatric disorder, history of COVID-19 infection, and month of baseline survey.  *The complete-case analysis of adjusted RR included 284 not vaccinated and 4,807 vaccinated individuals with two doses.  **+**The complete-case analysis of adjusted PR included 284 not vaccinated and 1,858 vaccinated individuals with one dose.  **The complete-case analysis of adjusted RR included 533 whose vaccination status was not reported and 4,807 vaccinated individuals with two doses.  ++The complete-case analysis of adjusted RR included 533 whose vaccination status was not reported and 1,858 vaccinated individuals with one dose. | | | | |
